# The Biophysical Interaction of the Danger-Associated Molecular Pattern (DAMP) Calreticulin with the Pattern-Associated Molecular Pattern (PAMP) Lipopolysaccharide

**DOI:** 10.3390/ijms20020408

**Published:** 2019-01-18

**Authors:** Unnati M. Pandya, Chinaza Egbuta, Trefa M. Abdullah Norman, Chih-Yuan (Edward) Chiang, Valerie R. Wiersma, Rekha G. Panchal, Edwin Bremer, Paul Eggleton, Leslie I Gold

**Affiliations:** 1 New York University School of Medicine--Langone Health, Departments of Medicine and Pathology, Division of Translational Medicine, 550 First Ave, New York, NY 10016, USA; unnatitauras99@gmail.com (U.M.P.); Chinaza.Egbuta@nyumc.org (C.E.); 2 University of Exeter Medical School, Exeter EX1 2LU, Devon UK; Ta314@exeter.ac.uk; 3 Target Discovery and Experimental Microbiology Department, Molecular and Translational Sciences Division, US Army Research Institute of Infectious Diseases, Frederick, MD 21702, USA; chih-yuan.chiang.ctr@mail.mil (C.-Y.C.); rekha.g.panchal.civ@mail.mil (R.G.P.); 4 Department of Hematology, University Medical Center Groningen, University of Groningen, 9713GZ Groningen, The Netherlands; v.wiersma@umcg.nl (V.R.W.); e.bremer@umcg.nl (E.B.); 5 UCB Pharma, Slough SL1 3WE, UK

**Keywords:** calreticulin, endoplasmic reticulum chaperone, innate immunity, lipopolysaccharide, Danger Associated Molecular Patterns (DAMPs), Pathogen Associated Molecular Patterns (PAMPs), bacterial opsonin

## Abstract

The endoplasmic reticulum (ER) chaperone protein, calreticulin (CRT), is essential for proper glycoprotein folding and maintaining cellular calcium homeostasis. During ER stress, CRT is overexpressed as part of the unfolded protein response (UPR). In addition, CRT can be released as a damage-associated molecular pattern (DAMP) molecule that may interact with pathogen-associated molecular patterns (PAMPs) during the innate immune response. One such PAMP is lipopolysaccharide (LPS), a component of the gram-negative bacterial cell wall. In this report, we show that recombinant and native human placental CRT strongly interacts with LPS in solution, solid phase, and the surface of gram-negative and gram-positive bacteria. Furthermore, LPS induces oilgomerization of CRT with a disappearance of the monomeric form. The application of recombinant CRT (rCRT) to size exclusion and anion exchange chromatography shows an atypical heterogeneous elution profile, indicating that LPS affects the conformation and ionic charge of CRT. Interestingly, LPS bound to CRT is detected in sera of bronchiectasis patients with chronic bacterial infections. By ELISA, rCRT dose-dependently bound to solid phase LPS via the N- and C-domain globular head region of CRT and the C-domain alone. The specific interaction of CRT with LPS may be important in PAMP innate immunity.

## 1. Introduction

Calreticulin (CRT) is a highly conserved embryonically lethal endoplasmic reticulum (ER) protein that is essential for quality control of protein folding and intracellular calcium homeostasis [1]. CRT mediates many physiological and pathological processes from within the ER, the cytoplasm, the cell surface and the extracellular space [2,3,4]. CRT is increased during ER stress and cellular injury as a coping mechanism that is important in identifying unfolded proteins. As a cell protection mechanism, CRT is involved in the unfolded protein response (UPR) [5,6,7] involving degradation of misfolded proteins or halting translation. A major role for CRT in the innate and adaptive immune response is exemplified by its requirement for phagocytosis and MHC Class I antigen processing by antigen presenting cells (APCs), respectively [8,9,10]. Specifically, CRT on the cell surface of apoptotic cells induces engulfment by professional (e.g., macrophages) and non-professional phagocytes. Moreover, CRT on the surface of cancer cells, induced by irradiation and chemotherapy, signals uptake by dendritic cells, referred to as immunogenic cell death (ICD) [2,11,12,13,14,15,16,17]. Originally, CRT was shown to be a C1q receptor on the cell surface of early apoptotic cells that bound to the globular region of C1q, thereby inducing phagocytosis followed by an immunogenic response, including cytokine release [4,18,19,20,21,22].

Based on its surface (re)localization during cellular stress, CRT typifies a danger-associated molecular pattern (DAMP) that is released in association with tissue damage or injury and in sensing pathogen-associated molecular patterns (PAMPs) via pattern recognition receptors (PRR) on cells of the innate immune system [2,23]. Microbial structures such as PAMPs are recognized by innate PRRs sensing receptors such as Toll-like receptors (TLRs) and an array of scavenger receptor class of proteins that initiate signaling cascades to promote inflammation, tissue repair, and elicit the innate immune response [23,24]. Recently, the PAMP, lipopolysaccharide (LPS), in a complex with the DAMP, HMGB1, was shown to trigger pro-inflammatory signaling that was distinct from LPS treatment or HMGB1 treatment alone in the context of sepsis [25]. For CRT, a reportedly endotoxin-free preparation induced the expression of cell surface maturation markers and cytokine release by dendritic cells via NFkB signaling [17]. Conversely, removal of endotoxin from CRT in other studies abrogated the upregulation of cell surface “activation” markers, such as MHCII and CD86, on dendritic cells and, pro-inflammatory cytokine production [26]. Similarly, following removal of LPS (to 0.01 EU/µg) from CRT using Concanavalin A, only ERK activation was maintained [27]. The mechanism underlying the immuno-stimulatory effects of CRT is currently unclear and may derive from direct CRT effects alone or may rely on the association with the PAMP, LPS.

Endotoxin (lipopolysaccharide) is the most prevalent lipid on the outer membrane of gram-negative bacteria, such as *E. coli*, and a potent inducer of immune cell activation, including phagocytosis, and release of cytokines mainly via binding to TLRs. The PRR, CD14, binds LPS via a 60 kDa LPS Binding Protein (LBP), which facilitates binding to the TLR complex for downstream signaling. The X-ray crystal structure of human CD14 defines an expanded amino terminal pocket that putatively binds acylated ligands such as LPS [28]. Whereas LPS boosts the immune response and is thus, considered to be protective of host immunity, miniscule amounts can cause septic shock. LPS is composed of a lipid A portion (responsible for its virulence) consisting of phosphorylated N-acetylglucosamine with a fatty acid side chain attached and two polysaccharide components (immunogenicity regions), O and R [29]. LPS tends to aggregate due its amphipathic, anionic molecular composition [30] and is a frequent contaminant of positively charged [cationic] proteins. LPS is notoriously difficult to remove from proteins and remains bound even after treatment with harsh organic solvents [31,32]. 

The molecular structure of CRT allows for its function in calcium sensing in the ER and its ability to identify carbohydrate moieties on glycoproteins used to assist in proper protein folding prior to exiting from the ER [33]. CRT is composed of 417 amino acids with a signal sequence in its N-terminus and a KDEL ER retrieval sequence at its C-terminus. The mature protein is divided into three domains with distinct molecular structures and functional activities [3,4,34,35,36]. For example, the function of calcium-dependent phagocytosis was shown to occur via different domains of CRT interacting with phosphatidyl serine (PS; C-domain) on apoptotic cells and a different domain (P-domain) bridging to a receptor and co-receptor on phagocytes [37]. Linearly, the N-domain (residues 18–197), composed of 12 beta sheets and two alpha helical structures, is followed by the proline-rich P-domain (198–308) that forms a paired hairpin structure containing high affinity calcium binding sites., Finally, the highly acidic C-domain (309–417) is mainly composed of one large alpha helix containing low affinity calcium binding sites. The X-ray crystal structure of the human CRT N-C globular domain has been solved, which shows an interaction of the N-terminal extension with the lectin binding site and reveals a peptide-binding site/cavity lined with hydrophobic residues [35]. CRT conformation is highly influenced by calcium concentrations within the ER and other locations causing flexibility of the P-domain loop, which impacts the peptide/lectin binding site for facilitating chaperone function [38]. 

Guided by our studies showing that human recombinant CRT (rCRT)-induced immune functions and the release of a variety of cytokines by mouse and human macrophages and that preparations of rCRT expressed in bacteria might harbor LPS, a potent inducer of similar immune responses [39,40,41], we performed the common Limulus Amebocyte Lysate (LAL) assay detection of LPS in our CRT preparations. This assay shows that human rCRT isolated from bacteria and yeast expression systems, as well as the native protein from human placenta, contain immune-function-inducible levels (by macrophages) of endotoxin (LPS). By immunoblotting, LPS was shown to induce oilgomerization of CRT. Furthermore, during both anion exchange and size exclusion chromatography, LPS affected both CRT binding and oilgomerization characteristics. In a solid phase assay, CRT directly bound to LPS in a dose-dependent manner, which was enhanced by calcium. Furthermore, CRT was shown to interact with LPS via its N-C globular head region (NC-domain lacking the P region) and separately, through its highly acidic C-domain. Interestingly, patients with bronchiectasis and underlying gram-negative bacterial infections show that CRT levels are high in the plasma and contain LPS bound to multimeric/oligomerized CRT. The physical binding interaction of CRT with LPS suggests a role for this interaction in DAMP-dependent PAMP immunity, such as enhancing innate immunity or preventing septic shock.

## 2. Results

### 2.1. Native Human and Recombinant Calreticulin and Calreticulin Domains are Associated with Endotoxin (LPS)

Recombinant human CRT (rCRT) preparations generated using diverse expression systems (bacterial, yeast, HEK293 cells) induced various immune functions in mouse and human macrophages (manuscript in preparation [41]). Lipopolysacchride (LPS) is known to similarly induce immune functions classically via Toll-like receptors (TLR) and other receptors on immune cells. To determine the amounts of LPS, a universal contaminant in bacterially expressed protein, the Limulus Amebocyte Lysate (LAL) assay, which recognizes the carbohydrate part of LPS, was used to test for possible contaminating endotoxin in the CRT preparations. As shown in Table 1, all preparations of purified CRT and CRT domains, regardless of source, harbored LPS ranging from 0.1 to 2.0 EU/µg of protein (*n* = 1–6). Notably, our studies show that 1.0 EU of LPS stimulates the release of an array of cytokines and activation of the NFkB pathway as observed by translocation from the cytoplasm to the nucleus in macrophages. In addition, 0.1 EU LPS potently stimulates granulocytes (manuscript in preparation [41]). An important point is that both yeast and native human CRT isolated from placenta and non-bacterial systems contained similar amounts of LPS that incurred during their respective purification procedures.

### 2.2. Calreticulin Associates With Certain Gram-Negative and Gram-Positive Bacteria

**Figure 1:**
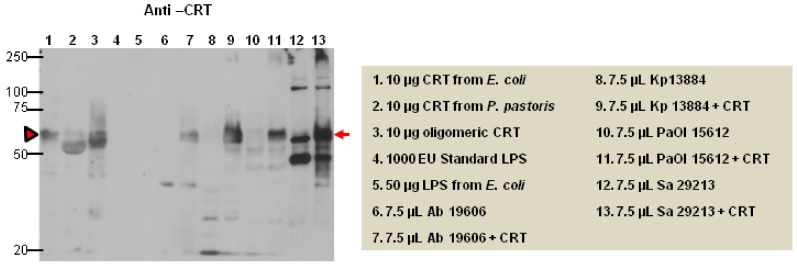
CRT binds to gram-positive and gram-negative bacteria. Recombinant CRT (rCRT) was incubated overnight at 4 °C with three gram-negative bacteria (*Acinetobactor baumannii* (Ab), *Psuedomonas aeruginosa* (PaOI), and *Klebseilla pneumonia* (Kp) and one gram-positive virulent bacteria (*Staphylococcus aureus* (Sa)). The cells were washed thoroughly after lysis and immunoblotted with anti-CRT (PA-3-900). The CRT antibody recognizes rCRT at ~63kDa (lane 1) from bacterial expression (red arrow head), as well as rCRT expressed in yeast (lane 2). Bacterially expressed rCRT (lane 1) contains an additional 23 amino acids at the N terminus (from pBAD plasmid gIII targeting CRT to the periplasmic space of bacteria) causing a slower migration than rCRT expressed in yeast (lane 2). CRT bound to both gram-negative and gram-positive bacteria (lanes 7, 9, 11 13) as shown as the same molecular weight as CRT alone (marked by a red arrow) *n* = 2.

The LAL assay suggests that CRT interacts with LPS and, hence, could potentially bind to LPS or peptidoglycans in bacterial cell walls. Therefore, CRT isolated from a bacterial expression system was incubated overnight at 4 °C with three gram-negative (*Acinetobacter baumannii, Psuedomonas aeruginosa, and Klebsiella pneumoniae)* and one gram-positive (*Staphylococcus aureus*) common virulent bacteria. The bacteria werewashed extensively and bacterial pellets with and without CRT were immunoblotted with anti-CRT. As shown in Figure 1 (red arrows), CRT bound to both gram-positive and gram-negative bacteria. In case of *S. aureus*, the anti-CRT antibody non-specifically immunoreacted with a minimal number of bands in the bacterial lysates (without CRT, lane 12), although there was a clear strong unequivocal interaction with CRT bound to the bacteria in the adjacent right lane (with CRT, lane 13). Of note, the bacterially produced rCRT contains an additional 23 amino acids in the N-terminus (lane 1) causing a slower migration than the yeast-produced rCRT (lane 2).

### 2.3. Lipopolysaccharide Induces Oilgomerization of Calreticulin and Simultaneously Decreases the Amount of the Monomeric Form

**Figure 2:**
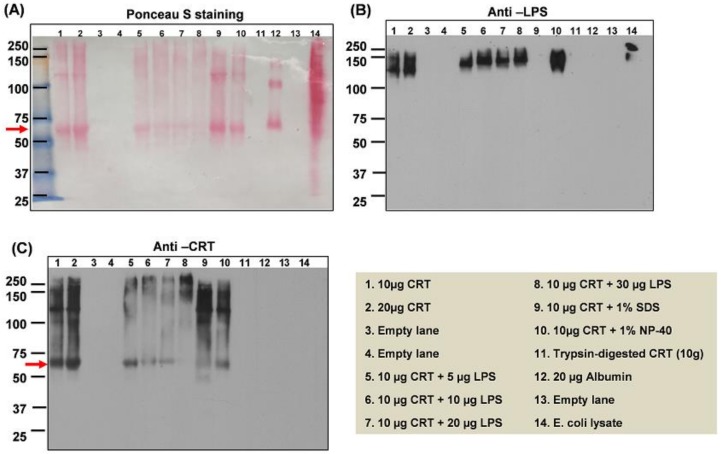
Immunoblot showing that LPS binds only to multimeric/oligomerized CRT and induces its oilgomerization. Recombinant CRT (rCRT) was mixed with increasing concentrations of LPS overnight at 4 °C, or 1% sodium dodecyl sulfate (SDS), 1% NP40, or trypsin (1:60 *w*/*w*) at 37 °C for 4 h. The samples were subjected to native gel electrophoresis followed by immunoblot analysis with anti-CRT or anti-LPS. (**A**) Ponceau Red; (**B**) anti-LPS; (**C**) anti-CRT. Increasing concentrations of LPS appear to induce multimeric forms of CRT while decreasing the CRT monomer (A and C, lanes 5–8). At 10 µg of CRT and 30 µg of LPS, there is little to no monomer remaining (A and C, lane 8). The detergents, 1% SDS (A and C, lane 9) and 1% NP-40 (A and C, lane 10) prevents CRT oligomerization. LPS remains bound to CRT in the presence of NP-40 but not in the presence of SDS, which removes all LPS from CRT (B, lane 9). Trypsin completely digested CRT (lane 11). LPS is only detected in high molecular weight aggregates of CRT (from ~130–240 kDA; lanes, 1, 2, 5–8, 10, 14). Specificity of the antibodies is ensured by the lack of detection of LPS (B, lane 12) or CRT (C, lane 12) with either antibody. The antibody to LPS only reacted with a high molecular weight form of LPS in the E. coli lysate (B, lane 14).

To determine the biochemical effects and binding interaction of LPS with rCRT in solution, increasing concentrations of LPS were added to CRT and the samples analyzed by native gel electrophoresis followed by immunoblotting separately, with antibodies to CRT and LPS. The Ponceau Red-stained membrane (Figure 2A) and immunoblot probed with anti-CRT antibodies (Figure 2C), show that without denaturation, CRT exists as a monomer at ~65 kDa (lanes 1 and 2; arrow), as dimers and oilgomerized forms of approximately 130–250 kDa. However, following the addition of increasing concentrations of LPS (5–30 µg) to 10 µg CRT, there is an increasing disappearance of the monomeric form of CRT (Figure 2C; lanes 5–8). Interestingly, as shown in Figure 2B, the blot immunoreacting with anti-LPS demonstrates that LPS only binds to oilgomerized CRT (lanes 1 and 2). Furthermore, as the monomeric form of CRT disappears, the oilgomerized forms increase (Figure 2C, lanes 5–8). Notably, CRT migration by immunoblot analysis shows a similar result with increasing CRT signal at higher molecular weights, suggesting that LPS induces multimerization/aggregation of CRT (Figure 2C; lane 8). 

To determine whether detergents could remove LPS from CRT, the rCRT samples were treated with the non-ionic and ionic detergents, Nonidet P-40 (NP-40) and sodium dodecyl sulfate (SDS), respectively. NP-40 did not affect LPS bound to oilgomerized CRT (Figure 2B, lane 10) whereas SDS treatment completely disrupted the interaction of LPS with CRT (Figure 2B, lane 9; compare with identical lanes in Figure 2C (anti-CRT)); NP-40 recovered the monomeric form of CRT, which migrated as the rCRT alone (Figure 2C, lane 10; compare with lanes 1 and 2). Trypsin (trypsin-to-protein ratio = 1:60 for 24 h) completely digested CRT with corresponding loss of LPS immunoreactivity (Figure 2B,C, lane 11). The *E. coli* lysate, as a positive control, immunoreacted with a higher molecular weight form of LPS (Figure 2B, lane14) than shown in the oilgomerized rCRT samples containing LPS (Figure 2B, lanes 1, 2, 5–8, 10). Albumin as a negative control (Figure 2B,C, lane 12) did not immunoreact with antibodies to LPS or CRT. Taken together, LPS induces higher order oligomerization of rCRT and since SDS disrupted this interaction, the binding between LPS and rCRT may in part be ionic by binding to negative phosphates on both the carbohydrate and Lipid A components of LPS. 

**Figure 3:**
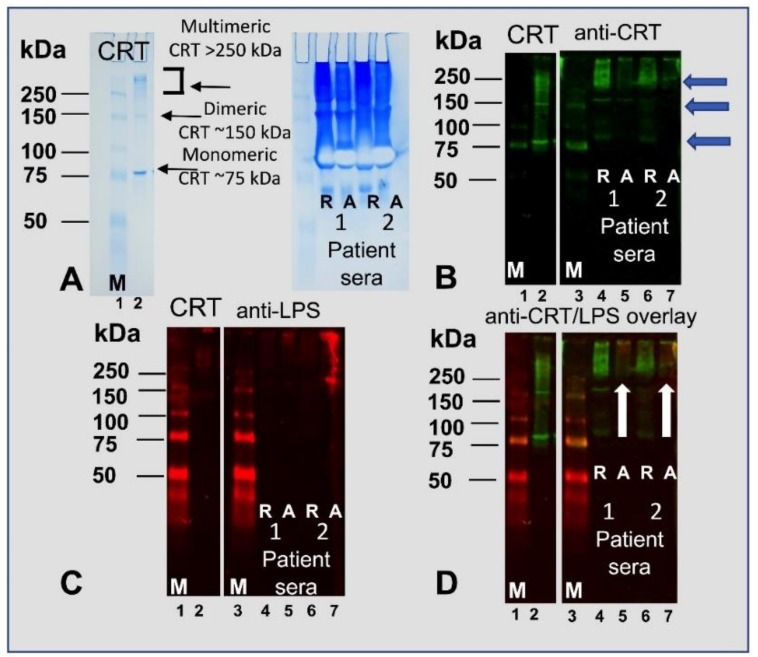
LPS is associated with higher molecular weight forms of calreticulin in gram-negative chronically infected bronchiectasis patients (**A**) CRT and two patient sera were run on a native PAGE gel under reducing [R] and alkylating [A] conditions and stained with Coomassie Blue and then immunoblotted with (**B**) anti-CRT (green; blue arrows depict varying molecular weight species of CRT), and (**C**) anti-LPS (red). (**D**) A double-probed immunoblot image using anti-human CRT antibody (green) and anti-*E. coli* LPS antibody (red) confirmed that LPS is associated with higher molecular weight aggregates of CRT. The white arrows indicate the association of LPS with CRT (yellow) in the two patient sera under alkylating conditions. Note: LPS does not bind to monomeric CRT in sera (C). The left panels in B, C and D show the immunoreactivity of the antibodies to rCRT and molecular weight markers (M, lanes 1 and 3) and rCRT in lane 2.

Higher median concentrations of CRT have been shown to circulate in blood plasma in pathological conditions such as rheumatoid arthritis (~10 ng/mL) and bronchiectasis compared to healthy subjects (2.9 ng/mL) [42]. Bronchiectasis patients commonly have chronic lung infections and serum levels of LPS may thus be elevated. Therefore, we assessed whether the binding interaction between CRT and LPS that we observe herein occurs physiologically in the serum of these patients [43]. The serum from four bronchiectasis patients that were known to have gram-negative bacterial infections were subjected to native gel electrophoresis and subsequently probed with antibodies to CRT and LPS. As shown in Figure 3A (right-hand lane), purified CRT (positive control for anti-CRT antibody) prepared in native sample buffer containing no reducing or alkylating agents separated as monomeric (~75 kDa) and multimeric complexes (150 kDa to >250 kDa). Sera from two patients that were both reduced (R) or alkylated (A) were run under the same native gel conditions and stained with Coomassie Blue. Figure 3B demonstrates that the anti-CRT antibodies detected both monomeric and high-molecular weight complexes of CRT in the sera of the two patients to varying degrees (green) except the alkylated samples were less immunoreactive likely due to modification of the epitopes. In Figure 3C, the sera from two patients contain LPS migrating at a high-molecular mass (red). LPS is shown bound to only oilgomerized forms of CRT migrating above 250 kDa but not to the ~75 kDa monomeric form (Figure 3D). Moreover, reducing the sera samples removed LPS from multimeric/aggregated CRT (Figure 3D, R) whereas alkylation retained the LPS on the CRT oligomerss (Figure 3D, A). Thus, the analysis using both rCRT produced in bacteria (Figure 3A-D; lane 2) and native CRT in patient serum (Figure 3A-D; lanes 4–7) illustrate that CRT can exist as oligomeric/aggregated and monomeric forms. However, the patient’s serum shows a greater amount of oilgomerized CRT (Figure 3, B–D, lanes 4-7). Therefore, the data suggests that CRT (Figure 3B, green) and LPS (Figure 3C, red) associate in serum as high molecular weight CRT–LPS complexes (Figure 3D; alkylated patient sera; lanes 5 and 7, yellow) suggesting that LPS binds to aggregates of CRT in the blood during pro-inflammatory conditions (or possibly in part induces CRT oligomerization).

### 2.4. Multimeric Forms of Human Recombinant Calreticulin are Demonstrated by Ion Exchange and Size Exclusion Chromatography

**Figure 4:**
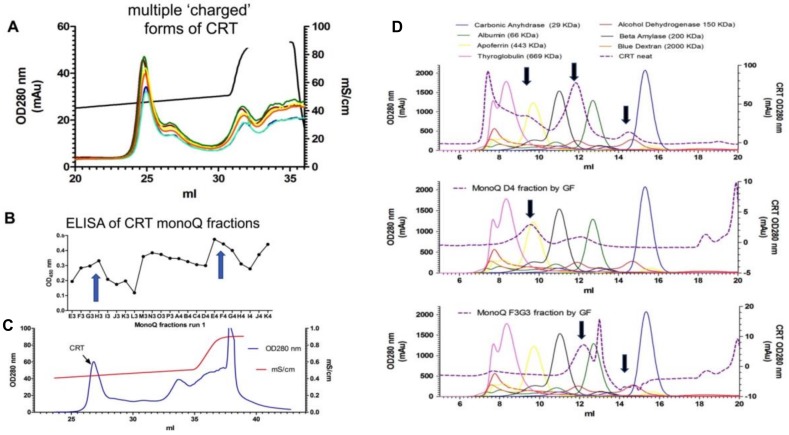
Chromatographic analysis demonstrating recombinant calreticulin forms various molecular complexes and charged complexes. (**A**) Multiple runs of purified recombinant CRT from *E. coli* (Intas) eluted from an anion exchange MonoQ column with 0–1 M NaCl. The first peak eluted at 240 mM NaCl, however a higher-charged complex elutes with >300 mM NaCl. (**B**) ELISA confirmed the various peaks were CRT and not contaminated proteins. The arrows depict the CRT content of the two major peaks shown in (A). (**C**) The two major peaks were pooled and re-run to confirm that the charge differences were retained. (**D**) Size exclusion chromatography showing elution of CRT (dotted line); CRT elutes as several molecular weight masses (black arrows) compared with standard proteins (top graph). Fractions D4 and F3G3 obtained from anion exchange MonoQ that were subsequently subjected to gel filtration confirmed that the purified protein fractions peak-D4 (middle) and fraction F3G3 (bottom graphs) were of different molecular masses of >443 and 60 kDa, respectively.

CRT is an acidic protein with an isoelectric point (pI) of 4.6 and therefore is classically separated by binding to anion exchange resin generally eluting at a concentration of >300 mM NaCl [34,36,44,45]. From our analysis above, LPS triggers higher order oligomerization of CRT. Therefore, we determined the charge and size characteristics of rCRT expressed in *E. coli* (used in experiments depicted in Figure 1, Figure 2 and Figure 3), shown to contain 0.3–1.2 µg/mL LPS (Table 1) by anion exchange (charge) and gel filtration (size) chromatography. Initially, rCRT was subjected to Q-Sepharose anion exchange chromatography and eluted with a NaCl gradient (0.02–1.0 M). As shown in Figure 4A, CRT eluted over a wide range of salt concentrations in multiple peaks, demonstrating that CRT exists in multiple forms, some of which have a weak negative charge while others have a relatively stronger negative charge. The main peak of purified CRT eluted from the anion exchange column at a higher salt concentration than expected (>300 mM NaCl), suggesting that the binding of negatively charged LPS to CRT increases its overall negative charge, thus requiring a greater salt concentration to remove the CRT–LPS complex from the column. Moreover, as there are multiple peaks, LPS–CRT complexes/aggregates (i.e., as shown in Figure 2 and Figure 3) exist with varying association affinities. Multiple fractions of the varied peaks of eluted protein were further confirmed to be CRT by direct ELISA (Figure 4B) and appeared only as one band under reducing conditions by SDS-PAGE stained with Coomassie Blue (not shown). The two major peaks from the ion exchange chromatography were pooled and subjected to a second anion exchange run (Figure 4C). Furthermore, during size exclusion analysis employing Superdex-200^®^ chromatography CRT eluted in the void volume as well as multiple peaks (Figure 4D), with the two major peaks appearing to correspond to ~450 and 60 kDa, respectively (Figure 4C). This suggests that CRT, at least in fluid phase, exists in both monomeric and multimeric complexes. 

**Figure 5:**
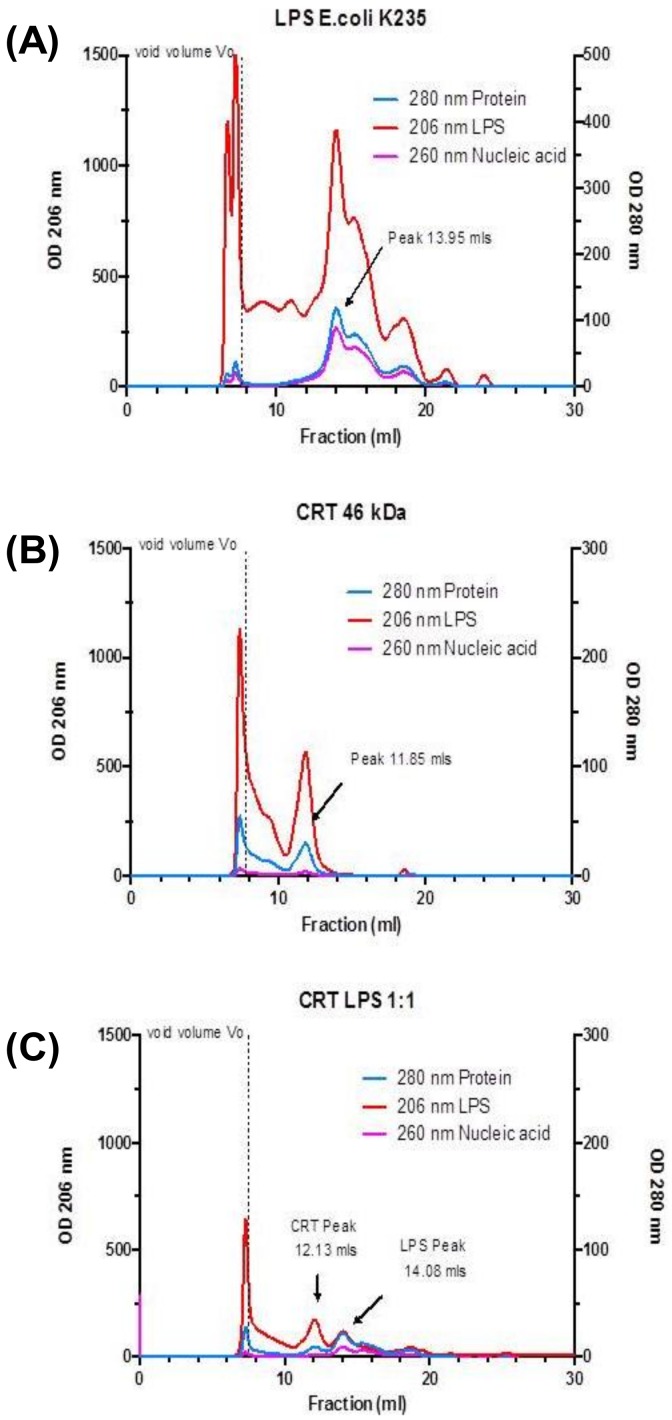
Size exclusion chromatography shows that CRT oligomerizes in the presence of LPS and contaminating LPS elutes with CRT. LPS (**A**), Purified recombinant calreticulin (**B**), or LPS mixed with CRT (1:1 *w*/*w*) (**C**) and incubated at 4 °C overnight were subjected to size exclusion chromatography (Superdex 200) in Tris buffered saline (TBS). LPS (red) already bound (contaminating) to CRT (middle graph) and additional LPS causes CRT (blue; lower graph) to elute in the column void volume and in multiple fractions through the internal diameter of the beads.

The molecular weight of CRT as determined by mass spectrometry is 46 kDa. However, by SDS-PAGE, reduced CRT migrates at an apparent relative molecular mass (M_r_) of 58–60 kDa putatively due to the CRT protein binding less SDS. Using a native PAGE gel, the M_r_ varies from 75 to 130 kDa. However, the mobility depends on both the protein’s charge and its hydrodynamic conformation/size. CRT under native conditions retains its elongated ‘comma-like’ conformation which slows its migration through the gel. To determine the effect of LPS on CRT size in fluid phase, LPS was combined with CRT and subjected to gel filtration chromatography compared with standard molecular weight markers. Initially, a gel filtration profile of LPS alone (Figure 5A) and CRT (Figure 5B) alone were generated. As expected, the commercially purified LPS consisted of a heterogeneous mixture of different fatty acid chain length species, as measured by absorbance at 206 nm wavelength; the dominant peak had a M_r_ of ~40 kDa. In addition, the LPS also has a 260 nm wavelength contaminating nucleic acid signature [46], which could be used to trace the LPS when mixed with CRT. The M_r_ of CRT when eluting through a gel filtration column is also approximately 110–130 kDa i.e., three times more than its calculated molecular weight. As shown in Figure 5C, the incubation of CRT with LPS (1:1), caused CRT to be retained and elute later. This was indicative of a change in the shape of CRT or joint mass when exposed to LPS of ~10 kDa molecular weight (mass). The elution place of CRT and CRT mixed with LPS from the column is as follows: CRT without additional LPS (elution volume: 11.85 ml = M_r_ 115 kDa; CRT–LPS 1:1 (elution volume: 12.13 mL = M_r_ 105 kDa). The elution profile of CRT combined with or without LPS from size exclusion chromatography shows that the conformation of CRT protein is altered in the presence of LPS. Possibly, the CRT becomes more compact and globular in the presence of LPS, allowing it to be retained longer on the column and elute later. 

### 2.5. Direct Physical Binding of CRT to LPS through the N-C Globular Head Region and the C-domain Determined by Solid Phase Assay

**Figure 6:**
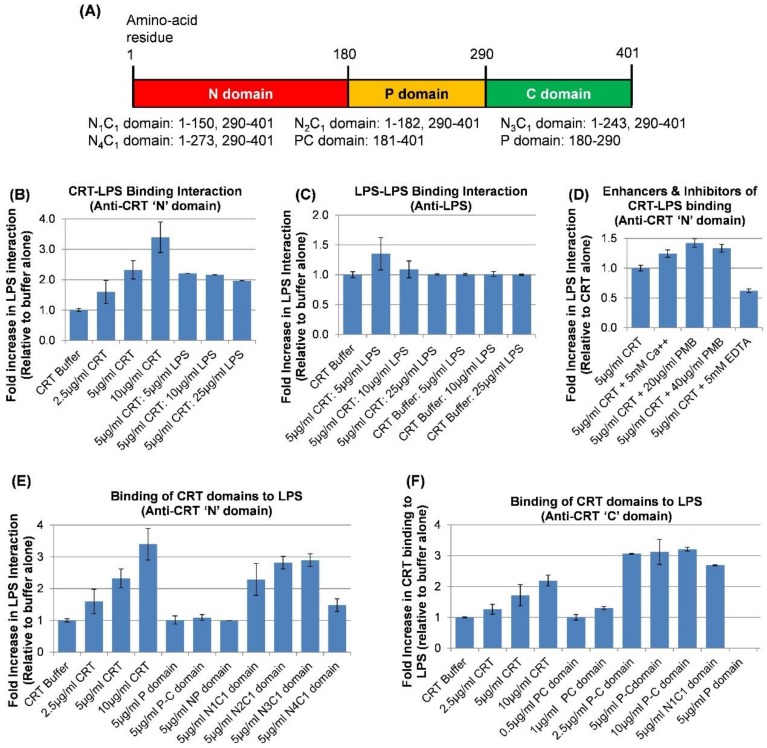
Direct ELISA assay showing the physical binding of calreticulin (CRT) to LPS. (**A**) Schematic showing CRT domains with respective amino acid numbering. The overlapping recombinant CRT (rCRT) domains (varying lengths of N-domain plus the C-domain) used in the ELISA are indicated below the diagram. Increasing doses of rCRT in Tris buffer in the presence or absence of different agents were incubated with immobilized LPS on an ELISA plate. (**B**) rCRT binds to immobilized LPS in a dose-dependent manner. The binding of rCRT to immobilized LPS is not due to the LPS already bound to CRT (as determined by LAL) since increasing concentrations of LPS (up to 25µg/mL) with 5.0 µg/mL CRT did not show greater binding to LPS than 5.0 µg/mL CRT alone; *n* = 6. (**C**) The addition of increasing concentrations of LPS in the presence and absence of rCRT does not increase the binding of LPS in solution to solid phase LPS as detected by an anti-LPS antibody; *n* = 3. (**D**) Calcium (5 mM) and polymyxin B (PMB; 20 µg/mL) increase the binding of CRT to LPS by 24% and 42%, respectively; *n* = 2. EDTA (5 mM) reduces CRT binding to LPS by 48%. (**E**) CRT appears to bind to LPS via its N-C globular head; the N1C1 fragment lacks the P-domain suggesting that the P domain is not necessary for CRT binding to LPS; *n* = 3. (**F**) Using an anti-C domain peptide antibody, the PC domain binds to LPS with three-times greater binding than the full-length CRT; *n* = 2.

To further determine whether human rCRT specifically binds to LPS, we developed a solid phase assay using 4.0 µg of Standard LPS from (K12 strain) coated in the wells of an ELISA plate. As shown in the graph in Figure 6B, following 18 h incubation with a dose range of CRT (2.5–10µg/mL), a dose-dependent increase in rCRT binding to LPS was obtained, suggesting the specificity of the binding interaction. The binding of CRT to LPS on the plate is not through the LPS associated with CRT since increasing concentrations of LPS (up to 25µg/mL) with 5.0 µg/mL CRT did not show greater binding to LPS on the plate than 5.0 µg/mL CRT alone. In addition, using anti-LPS antibodies, increasing concentrations of LPS added to the LPS-coated wells did not show increased binding (Figure 6C). 

The antibiotic polymyxin B (PMB) binds to LPS [47,48] obviating detection of LPS by the LAL assay. In an attempt to remove or block LPS on CRT, CRT was pre-incubated with increasing concentrations of PMB prior to adding to the LPS-coated ELISA plate. Interestingly, the presence of PMB dose-dependently increased CRT binding to LPS by at least 33% (Figure 6D). The addition of 2 mM calcium (CRT buffer contains 3 mM calcium; total 5.0 mM) to CRT increased the binding of CRT to LPS by 24% whereas the addition of EDTA reduced the binding to LPS by 48% (*n* = 3). 

To gain insight into the potential site(s) in the CRT molecule that bind(s) LPS, various lengths of the N-domain expressed as a fusion protein with the C-domain (Figure 6A) were incubated with the LPS-coated ELISA plate and compared for LPS-binding to intact rCRT on a weight basis. As shown in Figure 6E, the N_1_C_1_ fragment, which represents the solved crystal structure known as the globular head of CRT [35] lacking the P-domain (comprising residues 1–150 of the N-domain and 290–410 of the C-domain) bound to LPS on the plate to the same extent as the intact full length rCRT. Thus, apparently, the P-domain is not necessary for CRT to interact with immobilized LPS on the plate. Since the anti-N-terminal CRT antibody (to a peptide in CRT N-terminus) used in the above experiments did not implicate binding of the P-domain or PC-domains to LPS on the plate (Figure 6E), we confirmed by immunoblotting that both the PC-domains were not immunoreactive with the anti-N-terminal CRT antibody. Therefore, we used an anti-peptide antibody to residues 399 to 414 in the C-domain of CRT as the primary antibody in detecting rCRT and rCRT domains binding to LPS. This antibody was previously confirmed to detect the N1C1 and PC domains by immunoblotting (not shown). Note that the acidic C-domain cannot be expressed in bacteria without the P-domain. As shown in Figure 6F, whereas CRT, the N_1_C_1_, and the PC-domains bound to LPS as detected by the antibody, the P-domain binding did not immunoreact or was not bound. These results imply that CRT interacts with LPS via the N- and C-domains (the NC globular head). However, in the absence of a commercial antibody to the P-domain, we cannot conclude that the P-domain does not interact with LPS. The PC-domain bound to LPS in a dose-dependent manner with a three-fold greater binding than full length rCRT. A saturation of binding of the PC-domain was shown at 2.5 µg/mL (102 nM). Therefore, the NC-domain globular head of CRT and the C-domain contain binding sites that separately interact with LPS. 

## 3. Discussion

Calreticulin (CRT) has an important apparent role in both innate immunity and adaptive immunity specifically as a critical component of the (intracellular) peptide loading complex necessary for antigen presentation. However, its immune function in invertebrates is solely related to the innate response in non-specific host defense against pathogens, which includes the rudimentary mechanism of phagocytosis by primitive efferocytes [49,50,51,52]. Because of the overlapping immune functions we observed for recombinant CRT (rCRT) and lipopolysaccharide (LPS) and the goal of assigning structure-function relationships of the CRT molecule using overlapping recombinant CRT-domain sequences with specific immune-related functions, we explored the possibility that the rCRT produced in *E. coli* used in our assays (manuscript in preparation [41]) contained endotoxin levels (LPS) sufficient to contribute to host immune functionality in a variety of assays. As measured by the LAL assay and as shown in Table 1, regardless of the source of rCRT from yeast and bacterial expression systems, the CRT domains from bacteria and native CRT purified from human placenta, all preparations of CRT contained between 0.10 and 2.0 EU LPS/µg of protein. Thus, LPS was not only found in the bacterially expressed proteins but acquired during all purification processes. It is notable that 10 EU/ml (and in human = 5.0 EU/kg or 0.2EU/kg intrathecal) is an acceptable level of LPS in recombinant proteins for research and clinical purposes [53,54]. Moreover, a test for pyrogenic substances is not required for many health-related products including certain vaccines [53]. The study described herein was designed to gain insight into the biophysical interaction between human rCRT and LPS.

Our studies demonstrate that human rCRT binds to three different gram-negative and one gram-positive bacteria, suggesting that CRT could be a potential opsonin for bacterial clearance by immune cells, for example, in wounds in which CRT has been shown to potently enhance tissue repair [55,56,57]. Presuming that CRT is directly binding to LPS on gram-negative bacteria, lipoteichoic acid (LTA), which has been shown to be similar to LPS in the induction of cytokines via CD14 signaling [58,59], is the likely candidate constituent for CRT-binding to gram-positive *S. aureus*. Since the Lipid A portion of LPS is inserted in the phospholipid membrane of the bacterial cell wall, this orientation suggests that CRT might interact with the carbohydrate portion of LPS on gram-negative bacteria [30,48]. Similarly, rCRT from Chinese Mitten Crab (*Eriocheir sinensis*), Yesso scallop (*Patinopecten yessoensis*), and amphioxus (*Branchiostoma japonicum*) bound to different gram-negative and the gram-positive bacteria, *S. aureus*, in each study [49,50,52]. Moreover, LPS and an array of bacteria were able to upregulate Chinese Mitten Crab CRT mRNA and protein as a stress-induced innate anti-bacterial immune response and further, to mediate the uptake of bacteria by the respective macrophages in each of the invertebrate species studied. Interestingly, human rCRT could be substituted for recombinant *amphioxus* CRT, a basal chordate pre-vertebrate fish, in the phagocytosis of *E. coli* and *S. aureus* by fish macrophages [49]. These studies exemplify the evolutionary relevance for the role for CRT, as a PAMP in the recognition of danger signals (DAMPS) and in the clearance of microbial pathogens as a necessary primitive response that persists through to higher organisms. 

Stress-responsive molecular chaperones of the heat shock cytoplasmic type (HSP 27, 60, 70, 90, 110) and of the glucose-regulated proteins that reside in the ER (grp78, 94/gp96,170 and CRT) have been shown to have both potent pro-inflammatory and anti-inflammatory activities [39,40,60,61]. Similar to CRT, HSP 60 and grp94/gp96 and other chaperones bind tightly to LPS and have been shown to enhance the immunostimulatory effects of LPS. Therefore, it has been reasoned that HSPs might contribute to the in vivo recognition of gram-negative bacteria via LPS binding to modulate the immune response via TLR4 [62]. Chaperone proteins are highly active and important to processes involving the function of innate (e.g., phagocytosis, cytokine release) and adaptive immune response (e.g., peptide loading complex and antigen presentation). However, by binding to LPS and the same array of signaling receptors, for example, Hsp60, Hsp70, as well as CRT, has confounded a straightforward dissection of separate roles for chaperones in immune regulation from LPS [63,64,65,66,67,68,69,70] [39]. For example, HSPs have been shown to bind to many classes of scavenger receptors and LRP1 (CD91), and TLRs and to signal for different functions [71]. LPS also binds to different classes of scavenger receptors [24,39,60]. Added together with the immune function of recombinant chaperones tested in vitro potentially harboring LPS, the duplicative roles of chaperones and LPS have been difficult to separate. 

We show that LPS is bound to only to multimeric/aggregated forms of CRT and that addition of increasing amounts of LPS induced further oilgomerization of CRT while simultaneously decreasing the monomer until the monomer disappeared on the blot probed with anti-CRT antibodies. A series of reports showed that self-oligomerized full length and fragments of rCRT were up to 100-fold more potent in immune activities, including cytokine and NO release and macrophage phagocytosis, than monomeric or native CRT isolated from mouse liver [65,66,67,68,72]. Furthermore, an immune activation site was mapped to residues 150–230 (C-terminal part of N-domain and 2/3 of the P-domain) in the CRT molecule [68]. In these studies, various signaling mechanisms, including scavenger receptor binding, endocytic uptake and internalization of oligomers, and downstream MAPK and NF-kappaB signaling [65,66,68], were shown to be responsible for the immune-related activities. As native CRT purified from mouse liver did not oligomerize and showed far less to no activity in immune induction activity, it was proposed that Cys105 and Cys137 that form intramolecular disulfide bonds and the free SH on Cys163 participate in self-oligomerization, and that this does not occur with the native form that does not aggregate [68]. Mutated Cys163 did not prevent CRT aggregation in one report [73]. Biophysically, the size exclusion chromatographic studies reported herein support the existence of rCRT in multimeric/aggregated forms, which eluted as a single peak (Figure 5B, fractions 11–12) but also eluted as oligomers in the void volume. Similarly, the broad peak obtained by gradient elution of rCRT from anion exchange chromatography, as shown by ELISA and SDS-PAGE of eluted fractions, suggests that the LPS bound to rCRT induces various degrees of ionic binding to the negatively charged resin and/or that different conformational states of CRT as a result of LPS binding expose different parts of the protein for anion exchange for hydroxyl groups on the protein. In one microscale thermophoresis analysis, in which increasing concentrations of LPS were added to the identical human rCRT preparation used in the studies herein, the binding curve was shifted to the right, indicating that weaker binding of LPS to CRT occurs with increasing concentrations of LPS. This implies that there are less available binding sites for LPS (Supplementary Figure S1).

Increased amounts of CRT have been observed in rheumatoid arthritis patients, systemic lupus erythematosus, and other pathological conditions [22,42]. We show that CRT exists as a monomer, dimer and in aggregated forms (above 250 kDa) in the sera from two patients with chronic gram-negative bacterial infections associated with bronchiectasis, a chronic disease in which the bronchi are damaged by recurring inflammation and infection [43]. Interestingly, as shown by the overlapping signal on the immunoblot (Figure 3), LPS only bound to multimeric CRT in the blood of these patients; it is possible that LPS from bacterial infections induces higher molecular weight forms of CRT in serum, possibly for greater immune activation. The LPS was either removed or epitopes altered as the overlapping signal was lost following reducing the blood samples but not if the sample was alkylated and thereby preserving bound LPS. 

The specificity of the CRT–LPS interaction is supported by the dose-response effect observed by the solid phase assay with pure LPS bound on the ELISA plate (CRT at 10 µg/mL bound 3.5-fold over the CRT buffer control). That calcium = induced increased binding of CRT to LPS is likely due to the structural change in CRT induced by this cation [38] with possible exposure of additional interactive sites. Moreover, CRT as a regulator of calcium levels in the ER, contains both low capacity high affinity and high capacity low affinity calcium binding sites in its P- and C-domains, respectively. In addition, in the presence or absence of calcium, the resultant conformation of CRT dictates its vulnerability to limited trypsin digestion yielding different peptide fragments [74]. The antimicrobial peptide, PMB, enhanced CRT binding to LPS. The LAL assay detects LPS by binding to the carbohydrate portion of LPS when detecting LPS bound to proteins. PMB binds to LPS via its positive charge to the highly negatively charged LPS via phosphates both in the Lipid A and carbohydrate portions of the molecule [47,48]. This high affinity binding of PMB to LPS neutralizes its bactericidal activity and completely obliterates LAL detection of LPS. By light scattering, it was shown that PMB induces oilgomerization of LPS to facilitate macrophage phagocytosis but also causes LPS receptor binding and the induction of leakage of bacterial contents [47]. It is difficult to interpret the effect of PMB on CRT binding to LPS but since we show that CRT does not bind to immobilized LPS via the LPS bound to CRT, stronger ionic interactions may have increased binding. Similarly, the increase in binding of CRT to LPS in the presence of calcium and the fact the ionic detergent 1% SDS, but not NP40, removes LPS shown in native gel electrophoresis (Figure 3B, Lane 9) together, suggest, at least in part, an ionic interaction between CRT and LPS. It is interesting that contaminating LPS has largely been found tenaciously associated with positively charged (basic) proteins with high isoelectric points (n.b., unlike acidic CRT), such as histones, in which various means to remove the strongly bound LPS proved unsuccessful. In fact, whereas enzymes, phenol extraction, and acid treatment failed to remove bound LPS (for depyrogenation of proteins) from cationic proteins, poly-L-lysine treatment showed some success [75], as well as very harsh elution by reversed phase HPLC with acid mixed in organic solvents, which was strong enough to destroy the organic resin used to purify the proteins [32]. 

Likely, CRT would not bind LPS via its lectin binding site required for directing protein folding because the terminal sugar on N-linked carbohydrate chains is specific for recognition of a terminal glucose on the proteins [33]; LPS contains O-linked carbohydrate chains with a terminal galactose [76]. Using rCRT overlapping fragments, the data suggests that CRT interacts with LPS via its globular NC-domains, which, according to X-ray crystallographic studies, are in close proximity as the C-domain is inserted into the globular N-C-domain [35]. The P-domain does not appear to bet necessary for binding to LPS. The PC-domain showed an increase in relative binding to solid phase LPS compared to the intact full molecule. This suggests a sequence-specific potentially ionic interaction that might be partially masked by the native conformation of the intact CRT molecule. Nonetheless, apparently rCRT and native CRT that both contain LPS by LAL assay potentially associate with LPS via at least two separate sites on the intact molecule. 

The chaperone function of CRT and other chaperones appears to explain their roles as DAMPs, sensing danger and injury including from one of the most important PAMPs, LPS. CRT may help to sense and present PAMPs via PRRs to the innate immune system. For years, a plethora of studies has pervaded the literature, still leaving immune activities for chaperones difficult to resolve [39]. If an effort to remove contaminating LPS from proteins derived from expression systems is not undertaken, it is difficult to prove that the immune functions observed for CRT are not related to the LPS bound to CRT. Taking an unbiased approach on existing literature, there are reviews that show ample evidence for favoring the important role of CRT in immunoregulatory response including early cytokine release upon danger sensing [39,40,60,71]. A recent study showed that the release of CRT from dying cells, prior to the appearance of PS on the cell surface, binds to macrophages thereby inducing numerous immune activities including pro-inflammatory cytokines, macrophage polarization, and expression of CD40 and CD274, and cell migration [64]. Compelling evidence for CRT, Hsp90 and gp96 in the adaptive immune response including cross presentation of HSP-chaperoned proteins, secretion of cytokines and priming of helper T cells via LRP1 signaling on antigen presenting cells has been shown [69,70]. Important to the specific function of these three chaperones, each one phosphorylates different residues in the cytoplasmic domain of LRP1 eliciting different unique cytokine profiles and affecting different T cell subsets. In these experiments, the chaperone proteins were purified from mouse liver with undetectable levels of LPS. These studies were able to separate receptor specificity for chaperones in vivo, which was shown to only bind to LRP1 whereas LPS binds only to CD14/TLR4. Conversely, other reports testing the validity of CRT and GRP94/gp96 from porcine pancreas in immune functions showed that following removal of associated LPS, numerous immune responses were lost except ERK phosphorylation [27].

In conclusion, evidence for CRT and other chaperones acting as a major DAMP system interacting with PAMPs such as LPS together demonstrate dual immunoregulatory activities in terms of both enhancing the immune response and preventing sepsis [39]. Are the plethora of the known immune response activities enhanced by the binding of CRT to LPS (DAMPs to PAMPs), rather than either one as signaling agonists alone? Because we show that all preparations of rCRT and CRT isolated from human tissue contain bound LPS (by LAL), it is certain that CRT has a strong attraction for LPS (acts as a sink or sponge), which exists everywhere including in the blood of patients with infections and rheumatoid pathologies. Ample evidence exists for “clean” CRT and other chaperones to be potent immunoregulatory agents. Therefore, the physiological significance of CRT–LPS binding in enhancing the immune response is strong [40,71]. Preliminary studies herein show that the addition of 500 pg CRT/cell enhanced the binding of LPS (by FACs) to human neutrophils by 1.5-fold (Supplementary Figure S2; *n* = 2). It is established that pre-apoptotic cells can express cell surface CRT and this can act as an immunogen to enhance maturation of dendritic cells and induction of innate and adaptive immunity [2,77]. The ability of CRT to induce cell-mediated immunity is unknown. However, if CRT acts as an adjunct molecule binding to molecules such as LPS it may enhance activation of immune pathways via TLRs and other receptor mediated pathways. For example, a recent report shows that the chaperone HMGB1, both alone and together with LPS, induced pro-inflammatory responses that prevented sepsis [25]. However, because of the seemingly ubiquitous association of CRT and other chaperones with LPS, in vitro studies using exogenous preparations of CRT to investigate immune cell specific responses should ensure that preparations are completely devoid of LPS.

## 4. Materials and Methods

### 4.1. Antibodies, Recombinant Proteins and Reagents

The following antibodies were purchased from Abcam (Cambridge, MA, USA): mouse monoclonal anti-calreticulin (CRT) antibody (clone 2D7/1), mouse monoclonal LPS antibody (ab35654), and chicken polyclonal anti *E. coli* LPS antibody (#ab211144), and chicken polyclonal anti-C-terminal peptide antibody (ab2980). Rabbit polyclonal anti-CRT antibody (PA3-900 [anti-N-terminus]) and horseradish peroxidase-conjugated goat anti-mouse antibody 32430 were purchased from Thermo Fisher (Rockford, IL, USA). Mouse monoclonal anti-CRT antibody (FMC-75) was from Enzo Life Sciences (Farmingdale, NY, USA). Standard LPS (cat # tlrl-eklps) and Ultra-pure LPS (tlrl-peklps) were from Invivogen™ (San Diego, CA, USA). The following reagents and materials were from Sigma: Tween-20 (P2287), NP-40 (#I8896), SDS (L4509), Trypsin (T8658), Casein blocking buffer (B6429), Polymyxin B (P4932), and Nunc Immuno Microwell 96-well plates (M9410).

### 4.2. Limulus Amebocyte Lysate (LAL) Endotoxin Detection Assay

The amount of endotoxin, expressed as EU/µg of protein in CRT from different sources (bacteria, human placenta, and yeast) and recombinant CRT domains was determined by the LAL assay. LAL assay was performed at different doses for all preparations except the N-P-domain (1.0 µg) and values were expressed as EU/µg Endotoxin detection kits (#88282) and (#L00350) were purchased from Pierce™ (Rockford, IL, USA) and Genscript™ (Piscataway, NJ, USA) respectively. These kits were used exactly as per manufacturer’s instructions with reagents and materials provided in the kits. Endotoxin levels were determined by measuring the activity of proenzyme Factor C in the presence of a synthetic chromogenic peptide substrate that releases p-nitroaniline (pNA) after proteolysis to produce a yellow color that can be measured by reading the absorbance at 405 nm (Pierce) or 545 nm (Genscript). Samples were run in duplicate or triplicate and standard deviations from the mean were calculated for all samples. A standard curve for quantification of endotoxin in each sample was derived by plotting the absorbance of four standards on the *x*-axis and corresponding endotoxin concentration on the *y*-axis. 

### 4.3. Calreticulin and Calreticulin Domains

Human CRT was obtained from the following sources: (i) expressed in *Pichia pastoris* and a gift from Dr. Evaldas Ciplys and Dr. Rimantas Slibinskas (Vilnius University, Lithuania) [78]; (ii) purified from human placenta as described [79]; (iii) cloned, expressed, and purified from *E. coli*, as previously described [45] and purchased from Intas Pharmaceuticals Ltd. (Ahmedabad, India) using the same methodology. Recombinant CRT (rCRT) domains were cloned into the pBAD plasmid vector, expressed in *E. coli* BL21 (DE3) cells (Invitrogen), and purified with Ni-NTA affinity column chromatography following the manufacturer’s protocol (QIAGEN; Cat #30230). The rCRT from Intas Pharmaceuticals was used for all experiments. The CRT domains were a kind gift from Dr. Marek Michalak (University of Alberta, Edmonton, Canada). The domains consist of the following amino acid sequences of CRT: N1C1 (amino acids: 1–150 and 290–401), N2C1 (amino acids: 1–182 and 290–401), N3C1 (amino acids: 1–243 and 290–401), N4C1 (amino acids: 1–273 and 290–401), N-P (1–290), P (181–290), and P-C (181–417). All CRT and domains were prepared and stored in CRT buffer (0.01 M Tris, 3 mM Ca, pH 7.6).

### 4.4. Binding of Calreticulin to Bacteria

*K. pneumonia* (QC strain), *P. aeruginosa* (01 strain), *A. baumannii* (QC strain) and *S. aureus* (QC strain) cell pellets were suspended in Tris buffered saline (TBS; 20 mM Tris, 0.15 M NaCl pH 7.4) to a value of 1.0 absorbance unit at 600 nm wavelength and incubated with 20 µg of CRT. The mixture was incubated at 4 °C overnight, centrifuged, and washed 4 times with TBS. The bacterial/CRT samples were lysed in 50ul 1X laemmli buffer, boiled for 10 min, and 5 μL of each sample resolved by SDS-PAGE using a 4–20% gradient gel immersed in running buffer (25 mM Tris, 192 mM Glycine pH 8.3). Samples were electrophoresed at constant voltage of 70 V for 20 min and 100 V for 45 min, and then transferred to a PVDF membrane (GE Healthcare, #10600023) by wet transfer at 4 °C at 100 V for 60 min. The blot was washed in TBS-0.1% Tween (TBST), blocked with 5% milk TBST for one hour, and incubated with rabbit anti-human CRT (PA3-900; 1:1000) overnight at 4 °C followed by horseradish peroxidase-conjugated secondary goat anti-mouse (Thermo Scientific, #32430; 1:2000 in TBST). Membranes were developed using SuperSignal West Dura extended duration substrate kit (Thermo Scientific; #34075) followed by exposure to Hyblot CL X-ray films (Denville E3012). 

### 4.5. Binding of Lipopolysaccharide to Calreticulin Detected by Immunoblotting 

rCRT (concentrations determined by BCA assay kit (Pierce) assay) were subjected to SDS-PAGE electrophoresis under native conditions. In addition, complexes of 10 µg/mL CRT mixed with increasing concentrations of LPS (5–30 µg; Standard LPS, Invivogen) were incubated overnight at 4 °C before running on a native gel. CRT (10 µg/mL) was also treated with SDS or NP-40 at a final concentration of 1% or digested with trypsin (Sigma T8658) for 4 h at 37 °C in trypsin digestion buffer (50 mM Tris-Cl, pH 7.8) at a ratio of 1:60 (*w*:*w*) All complexes were subjected to native gel electrophoresis using non-denatured 10% acrylamide gel and non-denaturing sample buffer (62.5 mM Tris-HCL, pH 6.8, 25% glycerol, 1% bromophenol blue) and running buffer (25 mM Tris, 192 mM glycine) for 4 h at 70 V. The samples were transferred to a nitrocellulose membrane (Bio-Rad, cat# 1620112) using transfer buffer (2.03g Tris base, 14.4 g Glycine, 1L double-distilled and de-ionized water) at 70 V for 2 h. The membranes were stained with 0.5% Ponceau red, washed in TBS, followed by blocking for 1 h at room temperature with 5% milk in TBST. Membranes were incubated overnight at 4 °C with primary antibodies diluted in TBST, hybridized with anti-LPS monoclonal antibody (1:500, Abcam ab35654), developed and incubated with stripping buffer (1.5% glycine, 0.1% SDS, 1% Tween-20, pH 2.2) for 10 min, re-blocked with 5% milk, and re-blotted with anti-human CRT antibody (1:500, Enzo Life Sciences ADI-SPA-601) followed by horseradish peroxidase-conjugated secondary goat anti-mouse diluted in TBST (1:2000, Thermo Scientific 32430). Membranes were developed using SuperSignal West Dura extended duration substrate kit (Thermo Scientific; #34075) followed by exposure on Hyblot CL X-ray films (Denville E3012).

For the experiment showing LPS bound to CRT in patient sera, patient serum samples were electrophoresed under native gel conditions. Briefly, samples diluted in 25 mM Tris glycine buffer (pH 8.3) were prepared freshly in an equal volume of sample buffer (2 × strength, 62.5 mM Tris-HCl, pH 6.8; 25% glycerol; 1% Bromophenol Blue). Samples were loaded onto non-denatured mini-protean TGX precast gels in running buffer (25 mM Tris, 192 mM Glycine, pH 8.3) and electrophoresed at constant voltage of 75 V for 15 min and then 100 V for 35–45 min. Gels were washed and stained with Bio-Safe Coomassie stain (BioRad). For replicate immunoblots, duplicate gels were transferred to nitrocellulose using premade blotting kits (Bio-Rad) in a Turboblotter (BioRad) at 1.3 mA/25 V for 15 min. The blot was washed in phosphate buffered saline-Tween (PBS; 10mM Na_2_HPO_4_, 2.7 mM KCl, 137mM NaCl, 1.76 mM KH_2_PO_4_- 0.1% Tween) at 4 °C, incubated simultaneously with 1:1000 dilutions of rabbit anti-human CRT (PA3-900) and mouse monoclonal [Abcam 2D7/1] against *E. coli* LPS in 10 ml non-protein blocking buffer overnight (Pierce). After 4 washes in PBS-Tween, the blot was probed with two secondary infrared antibodies (red anti-mouse and green-anti rabbit) at 1:15,000 diluted in blocking buffer at RT for 1 h and then digitally imaged using a LiCor scanner (Odyssey CLx Imager).

### 4.6. Anion Exchange Chromatography and ELISA of Oligomeric CRT

Human rCRT derived from *E. coli* (100 µg) was suspended in 400 ul of Buffer A (50 mM Tris-HCl, 0.02 mM NaCl, 0.005 M K-EDTA) and injected onto the AKTA MonoQ (Q-Sepharose) HR 5/5 anion exchange column. The sample was subjected to gradient elution with Buffer A and Buffer B (50 mM Tris-HCl, 1.0 M NaCl, 0.005M K-EDTA) and peaks collected as a single eluate. Fractions eluting at higher salt concentrations were collected and further analyzed by ELISA. Ten microliters of each fraction were added to 90 μL of sodium carbonate buffer (pH 9.6) and coated onto a Nunc 96-well plate. The plate was incubated at 4 °C overnight and the next day, washed 3 times with PBST. t Two hundred microliters of casein blocking buffer was added to each well for 30 min at 37 °C, after which the plate was washed 3 times in PBST. Rabbit anti-human CRT (PA3-900) at a dilution of 1:2000 was incubated with each well for 1 h at 37 °C. The plate was washed 3 times with PBST and incubated with peroxidase-conjugated secondary goat anti-rabbit IgG at a dilution of 1:2000 for 1 h at 37 °C. One hundred microliters of tetramethylbenzidine (TMB) was added to each well and the plate was gently shaken at room temperature for 30 min in the dark. The reaction was terminated by adding 0.6 M sulfuric acid and the absorbance was read immediately at 450 nM. Certain fractions of CRT from MonoQ were subjected to size exclusion chromatography (Superdex 200) and compared with molecular weight standards eluting from the column, as described below.

### 4.7. Size Exclusion Chromatographic Analysis of CRT

Anion exchange and size exclusion chromatography was essentially performed as previously described [73,80]. Aliquots of recombinant CRT in Tris buffered saline pH 7.0 were incubated overnight at 4 °C at concentration of 0.1 mg/mL alone or in combination with 0.1 mg/mL or 0.5 mg/mL LPS (CRT–LPS ratio 1:1 *w*/*w*). Subsequently, 100 μL aliquots of CRT alone, LPS alone and the CRT–LPS mixture were separately injected and chromatographed by size exclusion using Superdex-200 pre-packed commercial column 10/300 GL with a flow rate of 1 ml/min on an AKTA FPLC system (GE Healthcare, Amersham, UK; separation between 600 kDa to 10 kDa). The progress of the protein through the column was monitored for absorbance at 280 nm, nucleic acid (contaminant of LPS purification) at 260 nm and LPS at 206 nm. The contaminating nucleic acid (260 nm) in the LPS sample but not present in purified CRT, acted as a tracer molecule to monitor the elution of the LPS alone and LPS bound to CRT. 

### 4.8. Binding of CRT to LPS by Solid Phase Assay (ELISA)

To detect direct physical binding of CRT and CRT domains to LPS, a direct ELISA was performed, essentially as described [52]. Standard LPS (80 µg/mL, approximately, 4,000 EU/well; Invivogen) diluted in endotoxin free water was added to a 96-flat bottom well plate (Nunc Immuno Microwell) and incubated over night at 37 °C. Subsequently, the plate was heated at 60 °C for 30 min and the wells blocked with 600 µg/mL BSA in TBS for 2 h at 37 °C. Prior to adding to the ELISA plate, increasing concentrations of CRT, CRT with different reagents and CRT domains (peptide fragments) were incubated overnight at 4 °C. The test samples were the following: CRT at 2.5 μg/ml (52 nM), 5 μg/ml (104 nM) and 10 μg/ml (208 nM) in CRT buffer (Intas Biopharmaceuticals), N_1_C_1_-domain of CRT (globular domain comprising residues 1–150 and 290–401; 5 µg/mL [165 nM] in CRT buffer, PC-domain of CRT (comprising residues 181–401; 2.5 μg/ml [102 nM], 5 μg/ml [204 nM] and 10 μg/ml [408 nM] in CRT buffer), CRT plus Ca^2+^ (5 μg/ml [104 nM] CRT plus 5 mM Ca^2+^), CRT plus EDTA (5 μg/ml [104 nM] CRT plus 5 mM EDTA), and CRT plus Polymyxin B (PMB) (5 μg/ml [104 nM] or CRT 10 μg/ml [208 nM] plus 15 μM or 30 μM PMB). The plate prepared with bound LPS was washed with TBS buffer 4 times each for 30 s, and 100 μL of the CRT samples prepared the day before were diluted to the concentrations above in TBS containing 0.1 mg/mL BSA and added to the wells. The plate was incubated for 3 h at room temperature, overnight at 4 °C, washed with TBS, and incubated with 100 µl rabbit polyclonal anti-human CRT primary antibody to an N-terminal peptide (PA3-900; 1:500) or a chicken polyclonal anti-human CRT C-terminal primary antibody *(Ab2980) at 37 °C for 2 h. Subsequently, the plates were washed again and incubated with peroxidase-conjugated secondary goat anti-rabbit IgG at 100 µl/well for 1 h at 37 °C followed by TMB at 100 µl/well, and the plate was gently shaken at room temperature in the dark for 30 min. The reaction was terminated by adding 0.6 M sulfuric acid and absorbance read immediately at 450 nM. Increasing concentrations of BSA were added to the plate as a control. Compared to CRT, calcium was used at 4.8 × 10^4^ molar excess and PMB at 147-fold and 295-fold molar excess. To determine the binding site/region on CRT that binds LPS, recombinant CRT domains, NP, N_1_C, N_2_C, N_3_C, N_4_C, and PC, were added following incubation overnight (shown in Figure 6A).

## Figures and Tables

**Table 1:** Calreticulin (CRT) and CRT domains from multiple sources contain endotoxin (lipopolysaccharide, LPS).

CRT Source	Endotoxin Level (EU/µg)
Recombinant CRT from *Escherichia coli*	0.5 ± 0.4 (*n* = 6)
Recombinant CRT from *Pichia pastoris*	0.1 ± 0.02 (*n* = 3)
Recombinant CRT from *Saccharomyces Cerevisiae*	1.3 (*n* = 1)
Native CRT from human placenta	2.0 (*n* = 1)
Recombinant CRT NP-domain	1.7 (*n* = 1)
Recombinant CRT N1C1 domain	1.3 ± 0.6 (*n* = 2)
Recombinant CRT P-domain	0.4 ± 0.5 (*n* = 3)
Recombinant CRT PC domain	1.3 ± 0.2 (*n* = 2)

Preparations of purified CRT and CRT domains from different sources contain LPS. The Limulus Amebocyte Lysate (LAL) assay was performed for endotoxin (LPS) detection using kits as described in Section 4. The levels of endotoxin were averaged from triplicates of each sample from the different sources. The number (*n*) of times each experiment was performed per preparation is shown. The values show standard deviation from the mean for the number of experiments performed on preparations from the different sources except if *n* = 1, where only the average of the triplicates is shown. The levels of endotoxin in the different preparations range from 0.1 to 2.0 EU/µg of protein.

## Supplementary Materials

Supplementary materials can be found at http://www.mdpi.com/1422-0067/20/2/408/s1.

## Abbreviations

CRT	Calreticulin
DAMP	Damage-associated molecular pattern
ER	Endoplasmic reticulum
HMGB1	High mobility group box 1
ICD	Immunogenic cell death
LAL	Limulus Amoebocyte Lysate
LPS	Lipopolysaccharide
MFI	Mean fluorescence intensity
MHC	Major histocompatibility complex
M_r_	Relative molecular mass
PAMP	Pathogen-associated molecular pattern
PBST	PBS with 0.1% tween
PBS	Phosphate-buffered saline
PS	Phosphotidyl serine
PMB	Polymyxin B
pI	Isoelectric point
rCRT	Recombinant CRT
SDS	Sodium dodecyl sulfate
TBST	Tris-buffered saline containing 0.1% Tween
TLR	Toll-like receptor
TMB	Tetramethylbenzidine
