# Supplementary material for: The Biophysical Interaction of the Danger-Associated Molecular Pattern (DAMP) Calreticulin with the Pattern-Associated Molecular Pattern (PAMP) Lipopolysaccharide
Source: Int J Mol Sci. 2019 Jan 18;20(2):408. doi: 10.3390/ijms20020408 (PMC6359024; doi:10.3390/ijms20020408)
Supplement: Supplementary file 1 [file ijms-20-00408-s001.pdf]

Supplementary Figure 1

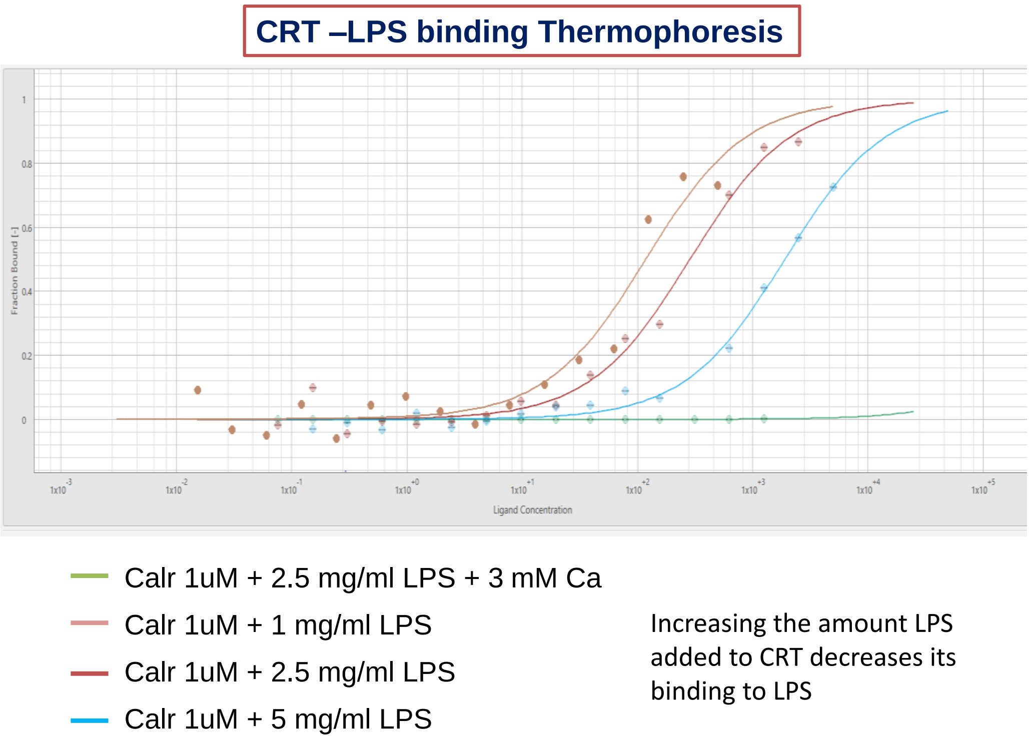

Supplementary Figure S1: Protein binding interaction between calreticulin (CRT) and lipopolysaccharide (LPS) by microscale thermophoresis (MST). Addition of increasing concentrations of LPS to recombinant CRT (rCRT) (harboring LPS as shown by LAL) decreases LPS affinity for CRT by MST. LPS was added at increasing concentrations (ranging from 2.5 mg/ml to 7 mg/ml) to a constant amount of N-hydroxysuccinimide (NHS) NHS–CRT. The NanoTemper system [1] was used to measure the binding of LPS to CRT. MST technology is based on the movement of molecules in a temperature gradient in which rapid measurements are recorded in solutions without requiring immobilization of one of the binding partners to a surface. Fluorescence changes of the fluorophore-labeled molecules are measured by the generation of a temperature gradient generated by an infrared laser in a micrometer-sized spot leading to the movement of the labeled molecules from a hotspot to the bulk solution (Soret coefficient). The microscale movements are followed by probing the time-dependent fluorescence changes in the hotspot [2,3]. The thermophoretic-induced migration of the fluorescent label is controlled by different parameters, such as the mass and shape of the moving fluorescent unit, and environmental effects, such as viscosity or ionic strength of the medium.

Purified rCRT was labeled with NT-647 dye (a reactive NHS-ester group that modified primary amines; NanoTemper Technology, Munchen, Germany) by N-hydroxysuccinimide (NHS) coupling, according to the manufacturer’s protocol. Increasing concentrations (2.5 mg/ml to 7mg/ml) of unlabeled LPS in MST buffer (20 mM HEPES, pH 8.0, 200 mM NaCl, 1 mM β-Mercaptoethanol, 0.05% Tween-20 with or without 3mM CaCl2) was added to a constant concentration of NT-647-labeled calreticulin (1 μM). Samples were loaded into 115 premium glass capillaries and MST analysis was performed using the Monolith NT.115 (NanoTemper Technology). The laser power was 20%. Data analysis was performed using NanoTemper Analysis software v.1.2.101. The results show a binding event between the fluorophore-labeled calreticulin and unlabeled LPS alters the mass of the moving constituent, which is reflected by changes in the thermophoretic time-dependent fluorescence curve that is related to the concentrations of the complexes. As the concentration of the LPS is increased, the changes in thermophoretic curve characteristics increase, implying the binding of the LPS ligand to calreticulin. In these experiments, calcium prevented the interaction between LPS and calreticulin.

1. Jerabek-Willemsen, M.; Wienken, C.J.; Braun, D.; Baaske, P.; Duhr, S. Molecular interaction studies using microscale thermophoresis. Assay Drug Dev. Technol. 2011, 9, 342–353.
2. Wienken, C. J.; Baaske, P.; Rothbauer, U.; Braun, D.; Duhr, S. Protein-Binding Assays in Biological Liquids Using Microscale Thermophoresis. *Nat. Commun.* **2010**, *1*, 100, DOI: 10.1038/ncomms1093
3. Entzian, C.; Schubert, T. Studying Small Molecule–Aptamer Interactions Using MicroScale Thermophoresis (MST). *Methods* **2016**, *97*, 27– 34, DOI: 10.1016/j.ymeth.2015.08.023

Supplementary Figure 2

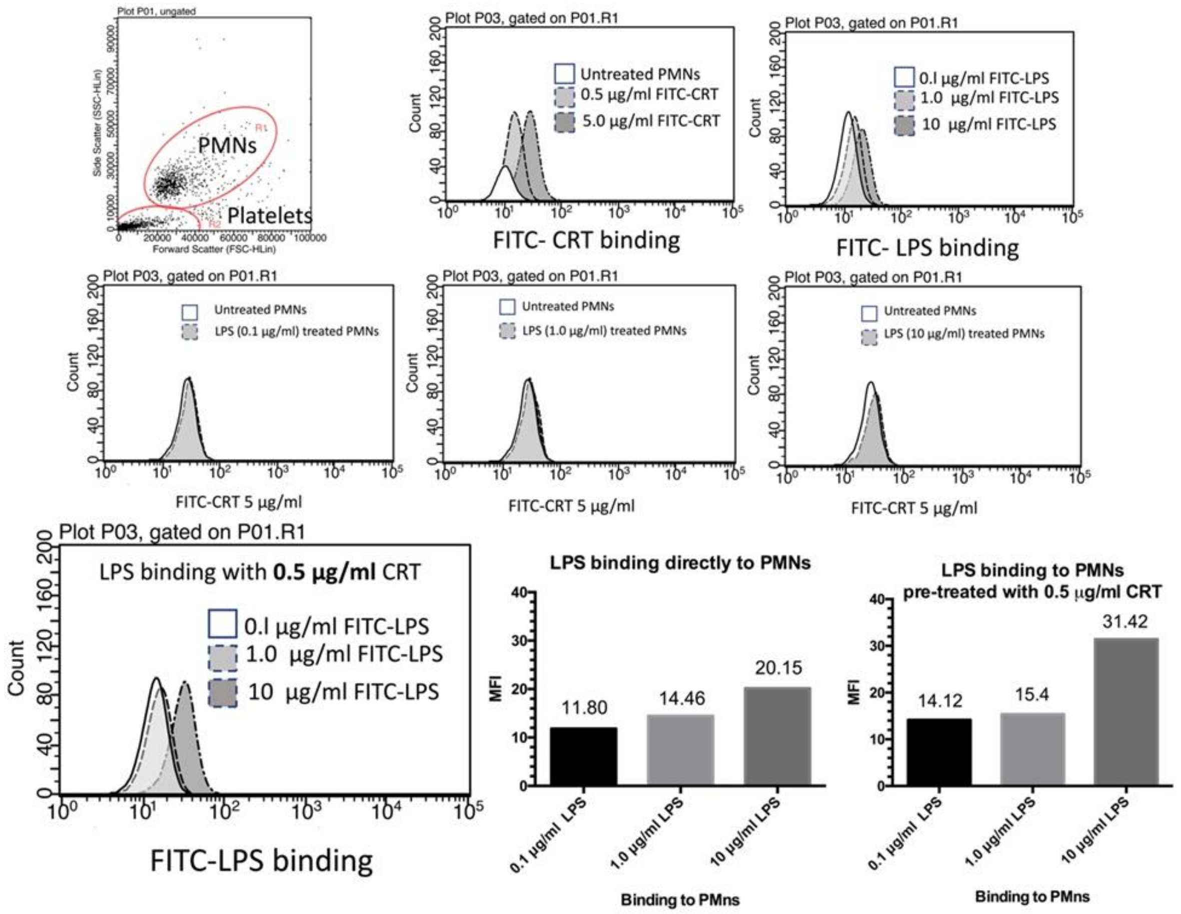

Supplemental Figure S2. Direct binding of fluorescein isothiocyanate (FITC)–CRT and FITC–LPS to human PMNs alone and in combination. PMNs were isolated from whole blood and incubated with either LPS and CRT alone to assess binding to neutrophils in a dose dependent manner (top panel). FITC-conjugated CRT did not bind to PMNs pre-treated with a range of LPS concentrations (middle panel). PMNs pre-treated with exogenous CRT enhanced the ability of LPS to bind to PMNs. Experiment shown is representative of three separate experiments.

Neutrophils were isolated from 50 ml EDTA-anticoagulated blood from two consenting adult volunteers to 95% purity using a previously described method [1]. Multiple aliquots of 100 µl of PMNs (5×10^5 cells) were added into microcentrifuge tubes, and the volume made up to 1 ml by adding 900 ul of RPMI. Fluorescein isothiocyanate (FITC)-labeled CRT or LPS were prepared according to the following method. Briefly, 0.5 mg CRT or 2.5 mg of LPS from *E. coli* K235 was suspended in 500 µl of sodium bicarbonate (pH 9.2), labelled with 50 µl of 3.0 mg/ml FITC prepared in the same buffer, and stirred for 2 h at room temperature in the dark at 4 °C. Free non-reacted LPS or CRT was removed by a Hitrap Sephadex G-25 5 ml desalting column using an AKTA FPLC system and fractions collected in 0.25 µl aliquots. The FITC-conjugated LPS or CRT elutes first from the column in the desired buffer and presents with a 280/495 nm absorbance spectrum confirming that the proteins are FITC-conjugated. Cell suspensions were treated with various concentrations of

unlabeled CRT or FITC–CRT (0.0, 0.5 μg/ml and 5.0 μg/ml final concentration) with and without LPS/ FITC–LPS (0.0, 0.1, 1.0 or 10 μg/ml final concentration) for 120 min at 37 °C. The cells were spun down at low speed (1500 rpm; 240× g) for 5 min and washed gently in PBS twice.

The effect of CRT on LPS binding or vice versa on human PMNs was assessed by flow cytometry using a Millipore Guava easyCyte instrument (Watford, Herts, UK) and the analysis was performed using GuavaSoft 3.1.1 Incyte® software. The upper panels show exogenous FITC–CRT and FITC–LPS to bind directly to PMNs. PMNs were gated for analysis as a purified population R1. It was demonstrated that FITC–CRT can bind directly to cells in a dose-dependent manner. Similarly, FITC–LPS also binds to PMNs directly. In addition, the ability of a fixed concentration of FITC CRT (5 μg/ml) to bind to PMNs untreated and treated with unlabeled LPS was assessed. The presence of LPS had no effect on the binding of CRT to PMNs. However, when cells were treated with unlabeled CRT (bottom panel) there was a 1.5-fold increase binding in 10 μg/ml LPS to PMNs compared to untreated cells. (MFI = 20.15 untreated cells; MFI = 31.42 in CRT treated cells; n = 2).

1. Suzanne Donnelly, Wendy Roake, Simon Brown, Philip Young, Haley Naik, Paul Wordsworth, David A. Isenberg, Kenneth B. M. Reid, and Paul Eggleton. Impaired Recognition of Apoptotic Neutrophils by the C1q/Calreticulin and CD91 Pathway in Systemic Lupus Erythematosus. Arthritis & Rheumatism Vol. 54, No. 5, May 2006, pp 1543–1556
